# How Not to Seek Promotion

**Published:** 1916-02-19

**Authors:** 


### THE MATRON'S AND SISTER'S DEPARTMENT.

### How Not to Seek Promotion.

A complaint as to the unbusinesslike ways of applicants for the post of assistant matrons, which will be found in the Editor's Letter-Box, is, it is to be feared, to some extent true. The fact is that the writing of business letters is not much in the line of ward sisters, from whose ranks the applicants for administrative posts are drawn. The hospital secretary who makes the complaint would probably find nothing unbusinesslike in the way these applicants prepare for an immediate operation or set to work to admit a number of wounded soldiers to their wards. In the five or six years that such an applicant has had charge of ward or theatre she has become methodical and neat to the highest degree in the work which she has mastered, but when confronted with a business letter, to the composing of which she is utterly unused, she fails miserably. It is a pity, because she will certainly be unsuccessful in obtaining the post which she desires, for which, after a few weeks of getting used to the work, she is probably well fitted; while at the end of a year's work in a matron's office she would conceivably refuse to believe that she ever could have been guilty of sending undated testimonials or of omitting to attach them to the application form. We think that if nursing sisters realised that, in addition to the detriment to themselves, how intensely annoying unbusinesslike habits are to others, they would, in spite of the scanty leisure at their disposal, resolve to grapple with the temptation to be careless even in the trivial correspondence of everyday life. They would be wise to make a rule never under any circumstances to send an undated letter, and set themselves resolutely to master the art of expressing themselves clearly and accurately on all, even unimportant, occasions. By preparing in this way the applicants for important posts will find that if they imagine themselves in the place of the prospective employer they will be able to give in a simple and concise way the information required.

